# Tenascin-C-Matrix Metalloproteinase-3 Phenotype and the Risk of Tendinopathy in High-Performance Athletes: A Case–Control Study

**DOI:** 10.3390/diagnostics14222469

**Published:** 2024-11-05

**Authors:** Lucas Rafael Lopes, Marcus Vinícius Galvão Amaral, Rodrigo Araujo Goes, Valéria Tavares, Francisca Dias, Rui Medeiros, Daniel Escorsim Machado, Jamila Alessandra Perini

**Affiliations:** 1 Research Laboratory of Pharmaceutical Science (LAPESF), Rio de Janeiro State University (UERJ), Av. Manuel Caldeira de Alvarenga, 1203—Campo Grande, Rio de Janeiro 23070-200, RJ, Brazil; lopes.rlucas02@gmail.com (L.R.L.); danielescorsim@yahoo.com.br (D.E.M.); 2 Program of Post-Graduation in Public Health and Environment, National School of Public Health, Oswald Cruz Foundation, Rio de Janeiro 21041-210, RJ, Brazil; 3 Research Division, National Institute of Traumatology and Orthopaedics, Rio de Janeiro 20940-070, RJ, Brazilrodrigogoes4@yahoo.com.br (R.A.G.); 4 Molecular Oncology and Viral Pathology Group, Research Center of IPO Porto (CI-IPOP)/Pathology and Laboratory Medicine Department, Clinical Pathology SV/RISE@CI-IPOP (Health Research Network), Portuguese Oncology Institute of Porto (IPO Porto)/Porto Comprehensive Cancer Centre (Porto CCC), 4200-072 Porto, Portugal; valeria.tavares@ipoporto.min-saude.pt (V.T.); ruimedei@ipoporto.min-saude.pt (R.M.); 5 Faculty of Medicine, University of Porto (FMUP), 4200-072 Porto, Portugal; 6 ICBAS—Instituto de Ciências Biomédicas Abel Salazar, Universidade do Porto, 4050-313 Porto, Portugal; 7 Research Department, Portuguese League Against Cancer (NRNorte), 4200-172 Porto, Portugal; 8 Faculty of Health Sciences, Fernando Pessoa University, 4200-150 Porto, Portugal

**Keywords:** tendinopathy, genetic polymorphism, TNC, MMP3, FBN2, athletes

## Abstract

**Background/Objectives:** Tendon structure is predominantly composed of the extracellular matrix (ECM), and genetic variants in non-collagenous ECM components may influence susceptibility to tendinopathy. We investigated the potential influence of single nucleotide polymorphisms (SNPs) in fibrillin-2 (*FBN2*), tenascin-C (*TNC*), and matrix metalloproteinase-3 (*MMP3*) on the tendon regeneration failure phenotype and impact on the susceptibility to tendinopathy in Brazilian high-performance athletes. **Methods:** This case–control study was conducted with 397 high-performance athletes from different sports modalities (197 tendinopathy cases and 200 controls), and they were analyzed by validated TaqMan^TM^ SNP genotyping assays of the SNPs *FBN2* (rs331079), *TNC* (rs2104772), and *MMP3* (rs591058). **Results:** Out of the 197 tendinopathy cases, 63% suffered from chronic tendon pain and 22% experienced more than three episodes of disease manifestation. The *TNC*-rs2104772-*A* allele was significantly associated with tendinopathy (OR: 1.4; 95% CI: 1.1–1.8), while athletes carrying the *MMP3*-rs591058-*T* allele were linked to an increased risk of more episodes of disease manifestation (OR: 1.7; 95% CI: 1.1–2.8). The *TNC-MMP3* tendon regeneration failure phenotype (*TNC-A/MMP3-T*) was associated with an increased risk of tendinopathy (OR: 1.4; 95% CI: 1.1–2.0) and episodes of disease manifestation (OR: 2.0; 95% CI: 1.2–3.5). Athletes with tendinopathy who had the *TNC-A/MMP3-T* interaction were more prone to experiencing more than three disease exacerbations (OR: 4.3; 95% CI: 1.8–10.5) compared to *TNC-A/TNC-C*. **Conclusions:** This study suggests that rs2104772 and rs591058 SNPs could be involved in the tendon regeneration failure phenotype and may influence the molecular mechanism related to the regulation of the tendon ECM during training workload.

## 1. Introduction

Tendinopathy is a common and complex disorder of the tendons with a particularly high prevalence among high-performance athletes (10–50%) [1,2]. The condition is characterized by abnormalities in tendon structure and involves inflammatory and angiogenic processes, leading to chronic pain and loss of strength and function [3,4,5,6,7]. Tendon structure is predominantly composed of the extracellular matrix (ECM), which includes collagenous and non-collagenous components to support its biomechanical activity [8,9]. Recent studies have shown that genetic variants, such as single nucleotide polymorphisms (SNPs), can influence the expression and function of non-collagenous components and play an important role in regulating the ECM, thereby altering the mechanical properties of tendons [10,11,12]. 

Fibrillin microfibrils play a critical role in providing strength and flexibility to the ECM. These structures are primarily composed of fibrillin-1 (FBN1) and fibrillin-2 (FBN2) [13]. The latter is associated with the formation of the structure of elastic fibers and is abundant in tendons [14,15]. The rs331079 (*G* > *C*) SNP in the *FBN2* gene (chromosome 5q23.3) is associated with Achilles tendinopathy susceptibility [12]. Another important protein regulating ECM elasticity is tenascin-C [16], a hexameric glycoprotein sensitive to mechanical loading during periods of high tendon stress [17]. The TNC protein is encoded by a gene of the same name (chromosome 9q33.1), and the *TNC* rs2104772 (*T* > *A*) SNP may affect the adaptive response in tendon remodeling to high-intensity exercise [18]. The rs2104772 SNP was found to have significantly upregulated tissue expression in injured tendons [19]. Remodeling of the tendon ECM is mediated by several enzymes, including members of the matrix metalloproteinase (MMP) family, to adapt to increased mechanical stress on tendon tissue [20]. The protein matrix metalloproteinase 3 (MMP3), also known as stromelysin 1, plays an important role in proteolytic activity, contributing to the degradation of collagen, proteoglycans, and glycoproteins to maintain ECM homeostasis [21]. However, the overexpression of MMP3 may contribute to pathological conditions leading to tendon degeneration [13]. The rs591058 (*C* > *T*) SNP in the *MMP3* gene (chromosome 11q22.3) is in linkage disequilibrium with other polymorphisms within or beyond this gene, which may affect gene expression and protein function, contributing to tendon ECM disorders [22]. Recently, the rs591058 SNP and other functional variants of the MMP3 gene were associated with non-contact anterior cruciate ligament (ACL) rupture in elite competitive athletes [23].

Given these implications, *FBN2* (rs331079), *TNC* (rs2104772), and *MMP3* (rs591058) SNPs may contribute to a more susceptible ECM tendon regeneration failure phenotype, and their interaction with environmental determinants may influence the individual risk for tendinopathy. Thus, the main aims of this study were to evaluate the effect of these SNPs and to determine whether there is a typical tendon regeneration failure phenotype that may contribute to susceptibility to tendinopathy in Brazilian high-performance athletes.

## 2. Materials and Methods

### 2.1. Study Design and Population

A case–control study was conducted with 397 Brazilian high-performance athletes recruited between February 2018 to June 2023 from different sports training clubs and competitions in the city of Rio de Janeiro. High-performance athletes were defined as professionals who receive a salary for their sport, participate in national and/or international competitions, have high oxygen volumes at anaerobic and aerobic thresholds, have advanced anthropometric and training measures (intensity, frequency, and duration), and have good running economy [24]. All participating athletes provided written informed consent and personally completed a self-reported questionnaire on their epidemiologic, sport, and clinical characteristics, including history of musculoskeletal injuries during their sports careers. The questionnaire was applied at the respective sports clubs, training centers, and/or medical facilities of each athlete. This questionnaire has been previously validated by experts in the field and is available online in a prior study [1]. At the end of the data collection, a trained observer reviewed the questionnaire with each athlete, and the database was completed by a trained researcher, with double verification conducted by different trained researchers. 

At the same site where the questionnaire was administered, samples of oral mucosal epithelial cells were collected with sterile swabs and stored in buffered solution, individually identified, and transported in an appropriate container, according to local safety regulations, to the Research Laboratory of Pharmaceutical Sciences—LAPESF (https://lapesfuerjzo.my.canva.site/#in%C3%ADcio, accessed on 31 October 2024) of the Rio de Janeiro State University (UERJ), in Rio de Janeiro-RJ, Brazil, which has adequate infrastructure for genetic analysis.

This study was approved by the Human Research Ethics Committee of the National Institute of Traumatology and Orthopaedics (protocol number 2.455.630/2017) and conducted in accordance with the Helsinki Declaration. The inclusion criteria encompassed Brazilian high-performance athletes, aged 18 to 45 years, participating in various sports, who self-identified as professionals, were affiliated with federations, and competed at the national and/or international level. Athletes without data on musculoskeletal injuries and/or those who did not provide sufficient biological material for analysis were excluded from this study. Athletes who self-reported clinically diagnosed tendinopathy by the specialized orthopedic surgeon were selected to compose the case group (*n* = 197) and provided specific information about the type, location, and episodes of disease manifestation. In the previously validated questionnaire [1], tendinopathy episodes were grouped in multiples of 3, allowing the athlete to select from the following options: no episodes, 1 to 3, 4 to 6, 7 to 9, 10 to 12, and more than 12. To understand the influence of SNPs on the molecular mechanism of disease recurrence, the current study considered 3 episodes as the cutoff point to avoid information and recall bias that could influence the association analysis with disease manifestation in athletes clinically diagnosed as cases. The control group (*n* = 200) consisted of athletes without evidence and reported incidents of musculoskeletal injuries.

### 2.2. Polymorphism Genotyping and Tendon Phenotypes

Genomic deoxyribonucleic acid DNA was obtained from oral mucosa collected from each athlete using the QIAamp^®^ DNA Mini extraction kit (Qiagen, Hilden, Germany) according to the manufacturer’s instructions. DNA concentration and purity were assessed using a Nanodrop^®^ spectrophotometer (Thermo Scientific, Wilmington, DE, USA). Genotyping analyses of *FBN2* (rs331079), *TNC* (rs2104772), and *MMP3* (rs591058) SNPs were conducted by allelic discrimination using TaqManTM SNP genotyping assays (Thermo Fisher Scientific, Waltham, MA, USA) (C_1561675_10, C_16182844_10, and C_785960_1_, respectively). SNP genotyping was made by a real-time polymerase chain reaction (PCR) using a 7500 Real-Time System (Thermo Fisher Scientific, Waltham, MA, USA), as previously described in a prior study [3]. 

Tendon phenotypes were assigned based on the *TNC* rs2104772 (*T* > *A*) and *MMP3* rs591058 (C > T) combinations, which comprised the following alleles at each locus of the chromosome: tendon stability (*TNC* allele *T* and *MMP3* allele *C*; *TNC* allele *T* and *MMP3* allele *T; TNC* allele *A* and *MMP3* allele *C*) and tendon regeneration failure (*TNC* allele *A* and *MMP3* allele *T*). In addition, the influence of *MMP3* rs59105-*C/T* alleles was compared in combination with the *TNC* rs2104772 allele *A* to verify which allele contributes more to the tendon regeneration failure phenotype, both in the development of tendinopathy in athletes and in the manifestation episodes of the disease.

### 2.3. Statistical Analysis

The sample size (n = 397) was appropriate to detect differences between the case and control groups, assuming an odds ratio (OR) of 2.0 with a power of 0.8 and 5% type I error, as calculated using Epi Info 7, version 7.1.3 (https://www.cdc.gov/epiinfo/support/downloads.html, accessed on 31 October 2024). The normality distribution was verified by the Kolmogorov–Smirnov test.

The variable Training Exposure Index (TEI) was created to estimate the volume and intensity of exposure to sports training in MET years. This was determined based on the years of training and the weekly training hours reported by each athlete, as well as the metabolic equivalent of the task according to the constants established for each sport in the 2011 Compendium of Physical Activities [25]. The continuous variables age, body mass index (BMI), years of training, weekly training hours, and TEI were not normally distributed (*p* < 0.01). Thus, comparisons of these variables between tendinopathy cases and control groups were performed using the Mann–Whitney test, and the data were presented as mean ± standard deviation (SD). However, according to their distribution and clinical significance, for the analysis, the continuous variables were categorized by the quartiles. The nominal data were shown in proportions, and differences between the two groups were evaluated using the Chi-squared (χ^2^) statistic test or Fischer exact test when applicable. Deviations from the Hardy–Weinberg equilibrium (HWE) of SNPs were assessed using the goodness-of-fit χ^2^ test. The distribution of alleles and genotypes of the *FBN2*, *TNC*, and *MMP3* SNPs was derived through gene counting, and the difference in frequencies between the two groups was evaluated using the χ^2^ test or Fisher’s exact test when appropriate.

The magnitude of the association between the presence of polymorphisms with tendinopathy and episodes of disease manifestation was estimated by OR, with their respective 95% confidence intervals (95%CIs), using a binary logistic regression model. The construction of the final model was based on the degree of statistical significance in the univariate analyses and on the biological importance of sociodemographic, lifestyle, sports, and training characteristics using the stepwise method with forward conditional selection. An input significance level of <0.20 (*p* < 0.20) was set, and the model retained variables with an output level of 0.05 (*p* < 0.05). The choice of the model was based on the quality of adjustment according to the Hosmer–Lemeshow goodness-of-fit test. All analyses were performed using the Statistical Package for Social Sciences (IBM Corp., Armonk, NY, USA).

## 3. Results

Out of the 197 tendinopathy cases, 79 (40.1%) presented the condition in the patellar region, 70 (35.5%) in the rotator cuff, 41 (20.8%) in the epicondyles, and 35 (17.8%) in the Achilles tendons. Moreover, 125 athletes (63.5%) suffered from chronic tendon pain, 43 (21.8%) experienced more than three episodes of disease manifestation, and 35 (17.8%) reported more than one tendon affected by the disease.

The athletes who comprised the case group were involved in various sports, including 72 (36.5%) in rugby, 35 (17.8%) in soccer, 28 (14.2%) in water polo, 22 (11.2%) in combat sports, 14 (7.1%) in handball, 7 (3.6%) in volleyball, 4 (2.0%) in basketball, 4 (2.0%) in football, 3 (1.5%) in artistic gymnastics, 2 (1.0%) in rowing, 2 (1.0%) in CrossFit, 2 (1.0%) in adapted rugby, 1 (0.5%) in swimming, and 1 (0.5%) in athletics. Meanwhile, the control group (n = 200) comprised 73 (36.5%) athletes in rugby, 50 (25.0%) in soccer, 23 (11.5%) in combat sports, 21 (10.5%) in water polo, 19 (9.5%) in handball, 3 (1.5%) in football, 3 (1.5%) in rowing, 3 (1.5%) in volleyball, 2 (1.0%) in CrossFit, 2 (1.0%) in swimming, and 1 (0.5%) in adapted rugby. There was no difference in the distribution of sports modalities between the two groups (cases and controls).

The comparisons of means between tendinopathy cases and controls were significantly different for age (25.8 ± 5.9 vs. 23.0 ± 5.0 years, *p* < 0.001), BMI (25.3 ± 3.6 vs. 24.3 ± 3.1 Kg/m^2^, *p* = 0.004), and TEI (9.9 ± 8.4 vs. 7.4 ± 6.9 MET years, *p* = 0.001). Table 1 describes the sociodemographic, lifestyle, sports, and training variables that entered the logistic regression model for analysis with tendinopathy presence. After the stepwise method, the variables age, sex, BMI, nutritional guidance, and post-training pain remained in this model (*p* = 0.63, Hosmer–Lemeshow goodness-of-fit test). On the other hand, for the analysis of episodes of disease manifestation, only tendon pain and TEI were considered confounding variables (*p* = 0.66, Hosmer–Lemeshow goodness-of-fit test).

**Figure 1:**
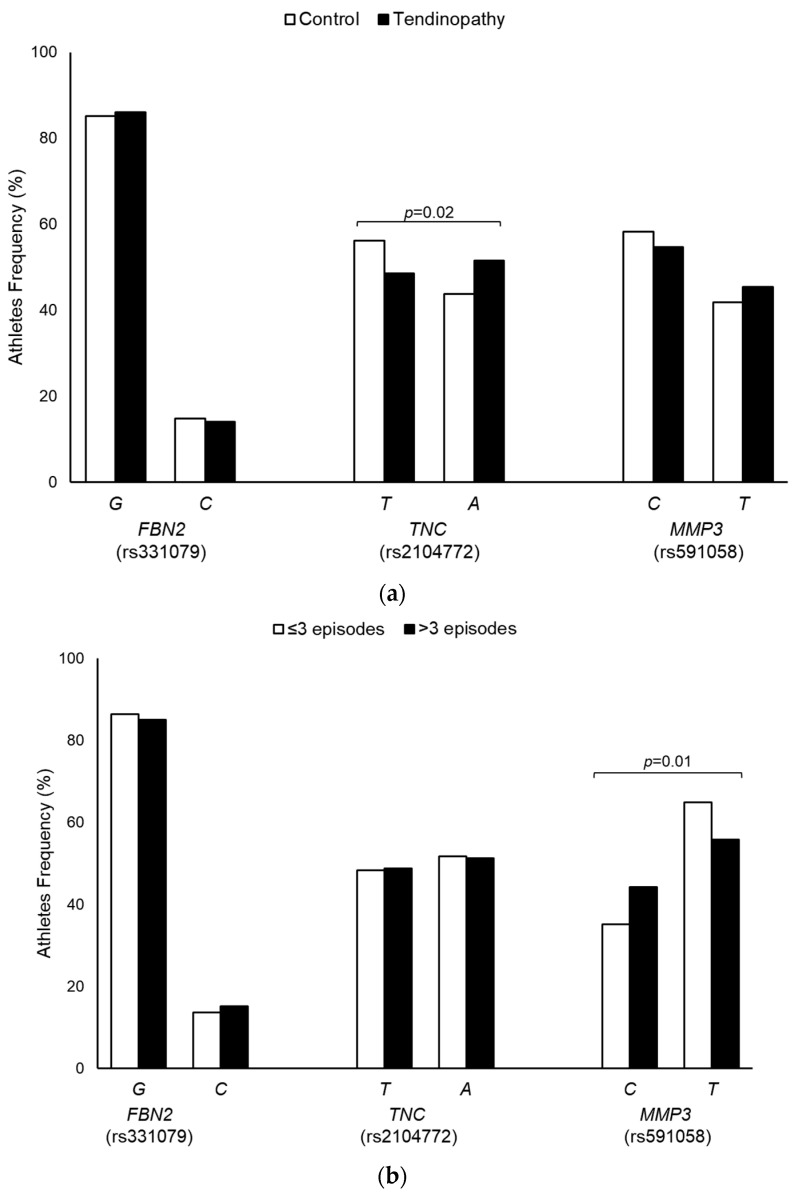
Allelic distribution of *FBN2*, *TNC*, and *MMP3* SNPs between controls and tendinopathy cases (**a**) and according to episodes of disease manifestation among tendinopathy athletes (**b**).

The *FBN2* (rs331079), *TNC* (rs2104772), and *MMP3* (rs591058) SNPs were in Hardy–Weinberg equilibrium in the overall participants athletes and in each group (tendinopathy cases and controls). The allelic frequencies of these SNPs are shown in Figure 1. Association analyses of *TNC* and *MMP3* SNPs with tendinopathy risk and episodes of disease manifestation are shown in Table 2. The presence of the *TNC*-rs2104772-A allele was associated with an approximately two-fold increased risk of tendinopathy in high-performance athletes. Similarly, *MMP3*-rs591058-T was associated with a four-fold increase in the risk of experiencing more than three episodes of disease manifestation. In addition, no significant associations were found between the *FBN2*-rs331079 SNP and the presence or manifestation of tendinopathy in the analyzed athletes.

**Figure 2:**
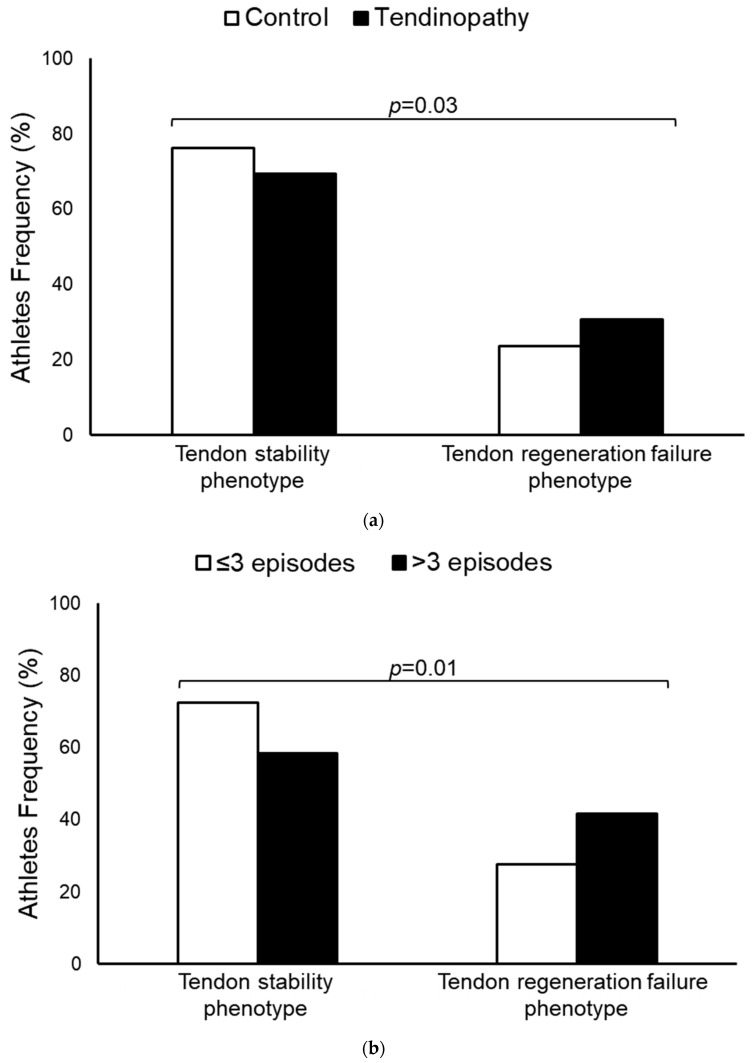
Tendon phenotype according to combinatorial analysis of *TNC* (rs2104772) and *MMP3* (rs591058) SNPs in the influence of tendinopathy development in athletes (**a**) and in the episodes of tendinopathy manifestation among high-performance athletes (**b**). Tendon phenotypes were classified based on *TNC-MMP3* combinations, including tendon stability (*TNC-T/MMP3-C*, *TNC-T/MMP3-T*, and *TNC-A/MMP3-C*) and tendon regeneration failure (*TNC-A/MMP3-T*).

The distributions of combination-predicted TNC-MMP3 phenotypes with tendinopathy risk and episodes of disease manifestation are shown in Figure 2. The tendon regeneration failure phenotype was associated with a 1.4-fold (95%CI: 1.1–2.0) increased risk for tendinopathy and a 2-fold (95%CI: 1.2–3.5) increase in the risk of experiencing more than three episodes of disease manifestation.

**Figure 3:**
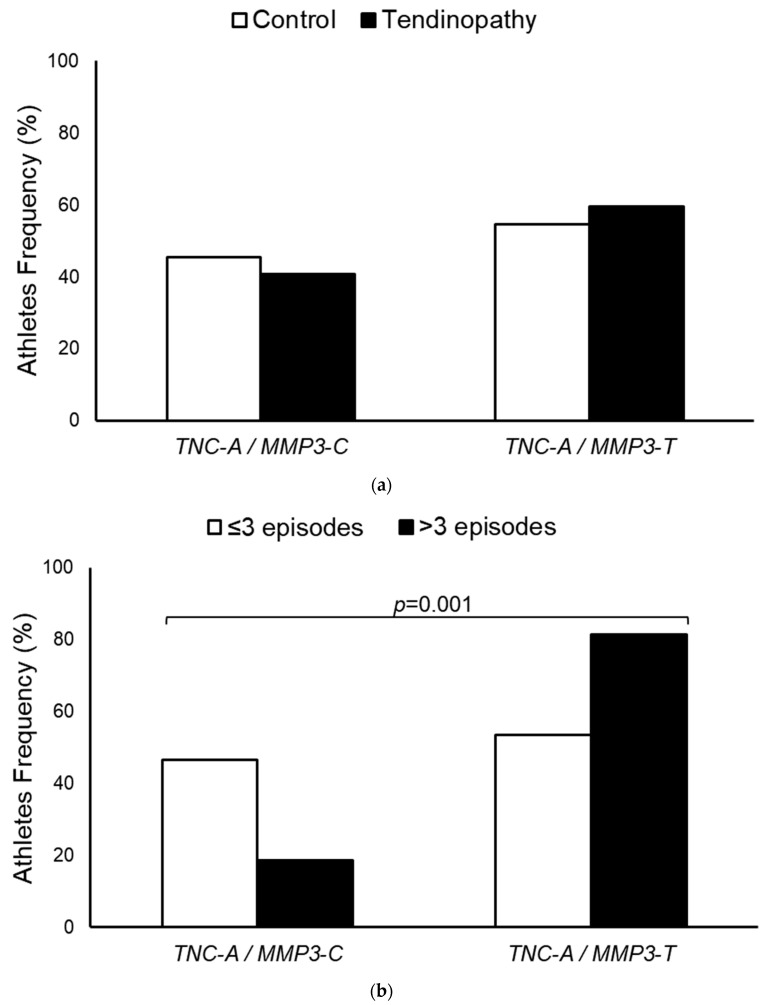
Influence of *MMP3* (rs591058) SNP among high-performance athletes with the presence of the *TNC-A* allele of rs2104772 SNP in the development of tendinopathy in athletes (**a**) and in the manifestation episodes of disease (**b**).

Additionally, tendinopathy case athletes with the *TNC-A/MMP3-T* profile had a 4.3-fold (95%CI: 1.8–10.5) higher chance of experiencing more than three disease exacerbations compared to those with up to three disease manifestations (Figure 3).

## 4. Discussion

This is the first study reporting on the influence of *TNC-MMP3* in the context of tendon regeneration failure phenotype, which is involved in the exacerbation of tendinopathy. SNPs in these genes are known to contribute to a post-transcriptional alteration in the regulation of genes involved in tendon ECM and damage to tissue regeneration [26,27]. The minor allelic frequencies (MAFs) of *TNC*-rs2104772-*A* and *MMP3*-rs591058-*T* are approximately 0.39–0.48 and 0.28–0.47, respectively, among different global populations (https://www.ensembl.org/index.html, acessed on 28 October 2024), which can contribute to approximately 30% of the studied athletes having a susceptibility to the tendon regeneration failure phenotype.

*TNC*-rs2104772 was associated with an increased risk (~2-fold) of tendinopathy in high-performance athletes, which corroborates the findings of other studies [19,28,29]. The TNC glycoprotein is composed of epidermal growth factor (EGF)-like repeats, fibronectin type III (FNIII)-like repeats, and a C-terminal fibrinogen-like globular domain. Interestingly, alternative splicing variants of *TNC* have an impact on cell surface receptors, including epidermal growth factor receptor (EGFR) [30]. Dejnek et al. (2022) evaluated 30 patients with lateral elbow tendinopathy who underwent a single autologous platelet-rich plasma (PRP) injection. Three months post-intervention, the concentration of EGF in the PRP from platelets demonstrated a significant correlation with improvements in grip strength, the strength of wrist extensors, and pain reduction [31].

The FNIII repeats influence the different isoforms of TNC glycoprotein [29]. The variant *TNC*-rs2104772-*T* consists of an exchange of the amino acid Leu1677Ile in the 13th FNIII domain within the beta-sheet structure of TNC, which results in steric hindrance with Phe1636 due to its side chain. This interference results in the larger isoform of TNC (230kDa), leading to increased instability [29] and affecting molecular elasticity [18,28], which may influence the function of this glycoprotein and negatively contribute to a failure in tendon ECM remodeling following stress caused by mechanical overload in high-performance athletes.

In addition, this study observed that the presence of the *MMP3*-rs591058-*T* allele was associated with a four-fold increased risk of the occurrence of more episodes of tendinopathy manifestations. The MMP3 is composed of the translocation signal peptide, propeptide, catalytic, and hemopexin domains [32]. The catalytic domain contains a Zn2+ binding sequence, which is responsible for the enzymatic activity of MMP3. Although the function of *MMP3*-rs591058 has not been fully understood, it is in linkage disequilibrium with other SNPs and forms functional haplotypes that influence MMP3 expression [21,33]. This variation may play a role in the degradation of tendon ECM molecules, including collagens, elastin, fibronectin, gelatins, laminins, and proteoglycans [32]. Brisk and colleagues observed that the rs591058-*TT* genotype, along with the contribution of the *T* allele on the haplotype with two other SNPs of *MMP3* (rs650108 and rs679620), indicated a predisposition to tendinopathies in high-level Croatian athletes [34], which is consistent with our findings.

The investigation of non-collagenous structures involved in tendinopathy has been relevant for understanding genotype–phenotype correlations in the non-remodeling of the ECM of injured tissue [27]. TNC and MMPs are co-expressed in tissues undergoing active remodeling in pathological conditions, suggesting mutual regulation [35]. Despite not finding an association with the *FBN2* SNP, this study observed a gene–gene interaction between *TNC-MMP3* and a potential tendon regeneration failure phenotype, where high-performance athletes presenting the *TNC*-rs2104772-*A* allele in combination with *MMP3*-rs591058-*T* had a higher (1.5 and 2.0, respectively) risk of tendinopathy and the occurrence of ≥3 episodes of disease manifestations. Moreover, the *MMP3*-rs591058-*T* variant demonstrated a four-fold increased risk of experiencing three or more episodes of disease manifestations compared to the SNP *C* allele in athletes carrying the *TNC*-rs2104772-*A* variant. TNC has been found to upregulate MMPs’ expression in synovial fibroblasts, resulting in the promotion of tissue remodeling [36]. This glycoprotein has two major isoforms (190 kDa and 230 kDa), and endurance training increased the content of the larger isoform in carriers of the *TNC*-rs2104772-*A* allele [37]. However, Siri and colleagues observed that the presence of the spliced sequence within the fibronectin-like type III repeats introduces new protease-sensitive sites in the large TNC isoform. Matriz metalloproteinase 2 (MMP2) and MMP3 exhibited proteolytic cleavage in approximately 60% of a single type III repeat of the large TNC isoform [38], which may lead to less tendon stability.

**Figure 4:**
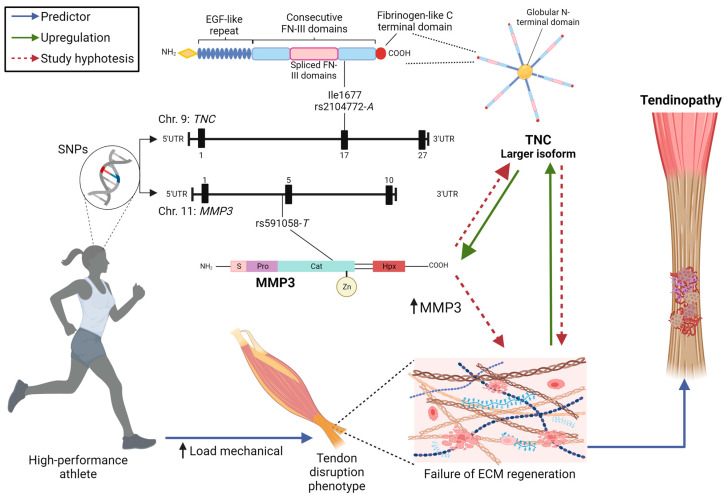
Hypothesis of the molecular mechanism involved by the *TNC*-rs2104772-*A*/*MMP3*-rs591058-*T* interaction in the failure of tendon regeneration after mechanical load during the sports career of high-performance athletes.

Finally, based on the associations found in the present study of the tendon regeneration failure phenotype with tendinopathy risk and higher episodes of disease manifestation, a hypothesis was created to describe the molecular mechanism by which *TNC*-rs2104772-*A*/*MMP3*-rs591058-*T* combination leads to the failure of tendon regeneration after mechanical load during the sports careers of high-performance athletes (Figure 4). In summary, individuals with the *TNC*-rs2104772-*A* variant allele present a larger isoform of TNC, and the increase in mechanical load positively regulates the expression of this larger glycoprotein isoform. TNC upregulates the expression of MMP3 for the regeneration of injured tissue; however, the influence of the rs591058-*T* allele in the spliceosome recognition region may intensify the expression and proteolytic function of this enzyme, leading to the degradation of the larger isoform of TNC. This can contribute to the reduced function of the cell adhesion-modulating ECM glycoprotein and failure in the regeneration of the tendon ECM during the training load exerted by high-performance athletes. Consequently, this mechanism leads to the development of tendinopathy and exacerbation periods of the disease throughout the athletes’ sports career.

Despite the promising findings, this study has some limitations that should be considered, including the small cohort size and the impossibility of conducting stratified analyses considering the dynamics and intensity of training in different sports modalities, each with its own profile for disease exacerbation. Furthermore, the sample size did not allow for stratification by affected tendon, as this would reduce statistical power. However, knowing that these SNPs are involved in the extracellular matrix of all tendon tissues, we believe that this hypothetical mechanism occurs in any tendon that is overloaded by excessive use due to athletic activity. We encourage the replication of this finding in other analytical studies with larger cohorts of high-performance athletes from different affected tendons and sports modalities to confirm the hypothesis of this study. Although the TEI was created to estimate the training load during the athletic career of each athlete, the MET used was based on a fixed value according to the 2011 Compendium of Physical Activities [25], which may not accurately reflect its true value. Additionally, there is a risk of recalled information and memory bias, as some athletes may not have remembered important details in the self-reported questionnaire. However, possible confounding variables were inserted in the logistic regression model to evaluate the real influence of SNPs on the development of tendinopathy.

The results of this study may have important implications for predicting and preventing the development of tendinopathy in elite athletes. The use of genetic testing in sports medicine can contribute to precision medicine programs by identifying variants that may increase the risk of injury and by designing personalized training programs for athletes to prevent the development of the disease. The identification of the TNC-rs2040772 and MMP3-rs591058 SNPs, along with other non-genetic predictors of disease (age, sex, training exposure time, and sport modality), could contribute to the development of complex risk assessment models (RAMs) to promote a better quality of life for more vulnerable athletes without compromising their athletic performance and mitigating the premature end of their careers.

## 5. Conclusions

In summary, high-performance athletes with *TNC*-rs2104772-*A* have a higher risk of developing tendinopathy, while *MMP3*-rs591058-*T* is associated with an increased risk of having more episodes of tendinopathy manifestations. The *TNC*-rs2104772-*A*/*MMP3*-rs591058-*T* combination suggests a phenotypic profile of tendon regeneration failure associated with the failure of tendon ECM regeneration, contributing to susceptibility and increased exacerbation of tendinopathy. Therefore, athletes at higher risk of developing tendinopathy could be identified by genetic testing for these SNPs.

## Figures and Tables

**Table 1:** Logistic regression model of the sociodemographic, lifestyle, and sports characteristics of study population (*n* = 397).

Characteristics	Control (*n* = 200)	Tendinopathy (*n* = 197)	*p*-Value ^a,b^	Crude ORs (CI 95%)	Adjusted ORs (CI 95%) ^b^
**Age (years) ^d^**	***n* (%)**				
≤23	124 (62.0)	79 (40.1)	<0.01	1 ^c^	1 ^c^
>23	76 (38.0)	118 (59.9)		2.44 (1.63–3.65)	**2.10 (1.37–3.23)**
**Sex**					
Female	67 (35.5)	85 (43.1)	0.03	1 ^c^	1 ^c^
Male	133 (66.5)	112 (56.9)		0.66 (0.44–0.99)	**0.60 (0.38–0.95)**
**BMI (Kg/m^2^) ^e^**					
<24.9	131 (66.5)	105 (53.3)	<0.01	1 ^c^	1 ^c^
≥25.0	66 (33.5)	92 (46.7)		1.74 (1.16–2.61)	**1.82 (1.16–2.88)**
**Skin color ^f^**					
White	70 (36.5)	94 (47.7)	0.18	1 ^c^	1 ^c^
Non-white	122 (63.5)	103 (52.3)		0.63 (0.41–0.94)	0.74 (0.47–1.15)
**Schooling ^g^**					
University	85 (42.9)	121 (61.4)	0.23	1 ^c^	1 ^c^
High-school	113 (57.1)	76 (38.6)		0.47 (0.32–0.71)	0.75 (0.47–1.20)
**Income familiar ^h^**					
>BRL 10.000	47 (24.1)	70 (36.3)	0.09	1 ^c^	1 ^c^
≤BRL 10.000	148 (75.9)	123 (63.7)		0.56 (0.36–0.87)	0.65 (0.40–1.06)
**Alcohol consumption**					
No	96 (48.0)	71 (36.0)	0.17	1 ^c^	1 ^c^
Yes	104 (52.0)	126 (64.0)		1.64 (1.10–2.45)	1.36 (0.87–2.11)
**Smoking**					
No	190 (95.0)	178 (90.4)	0.13	1 ^c^	1 ^c^
Yes	10 (5.0)	19 (9.6)		2.03 (0.91–4.48)	1.90 (0.82–4.40)
**Nutritional guidance**					
No	110 (55.0)	70 (35.5)	<0.01	1 ^c^	1 ^c^
Yes	90 (45.0)	127 (64.5)		2.22 (1.48–3.32)	**2.24 (1.45–3.47)**
**Continuous medication use ^f^**					
No	173 (90.1)	163 (82.7)	0.74	1 ^c^	1 ^c^
Yes	19 (9.9)	34 (17.3)		1.90 (1.04–3.46)	1.12 (0.57–2.21)
**Post-training pain**					
No	135 (67.5)	93 (47.2)	<0.01	1 ^c^	1 ^c^
Yes	65 (32.5)	104 (52.8)		2.32 (1.55–3.49)	**2.07 (1.34–3.20)**
**TEI (MET years) ^d,i^**					
≤7.2	120 (60.0)	87 (44.2)	0.02	1 ^c^	1 ^c^
>7.2	80 (40.0)	110 (55.8)		1.90 (1.27–2.83)	**1.67 (1.05–2.65)**

BMI: body mass index, CI: confidence interval, ORs: odds ratios, TEI: Training Exposure Index. ^a^ *p* ≤ 0.05 was obtained through the Chi-square test (Pearson’s *p*-value). ^b^ OR adjusted by age, sex, BMI, nutritional guidance, post-training pain, TEI. ^c^ Reference group. ^d^ Continuous variable was categorized by the median. ^e^ Information obtained from 394 athletes (control = 197). ^f^ Information obtained from 389 athletes (control = 192). ^g^ Information obtained from 395 athletes (control = 198). ^h^ Information obtained from 388 athletes (control = 195 and tendinopathy = 193). ^i^ Information obtained from 388 athletes (control = 197 and tendinopathy = 191).

**Table 2:** Association analysis of the *TNC* and *MMP3* SNPs between controls and tendinopathy cases (*n* = 397) and episodes of disease tendinopathy among cases (*n* = 197).

**SNPs**	**Control (*n* = 200)**	**Tendinopathy (*n* = 197)**	***p*-Value ^a,b^**	**Crude ORs** **(CI 95%)**	**Adjusted ORs** **(CI 95%) ^b^**
***TNC* ^d^** **rs2104772 (*T* > *A*)**	***n* (%)**				
TT	61 (30.5)	46 (23.5)	**0.04**	1 ^c^	1 ^c^
TA	103 (51.5)	98 (50.0)		1.26 (0.79–2.02)	1.37 (0.81–2.29)
AA	36 (18.0)	52 (26.5)		1.91 (1.08–3.39)	**2.22 (1.18–4.15)**
TA + AA	139 (69.5)	150 (76.5)	**0.03**	1.43 (0.91–2.24)	**1.80 (1.07–3.04)**
***MMP3* ^e^** **rs591058 (*C* > *T*)**					
CC	62 (33.0)	59 (29.9)	0.78	1 ^c^	1 ^c^
CT	95 (50.5)	97 (49.2)		1.07 (0.68–1.69)	1.04 (0.63–1.70)
TT	31 (16.5)	41(20.8)		1.39 (0.77–2.50)	1.24 (0.66–2.33)
CT + TT	126 (67.0)	138 (70.1)	0.72	1.15 (0.75–1.77)	1.09 (0.68–1.74)
**SNPs**	**Tendinopathy**		***p*-Value ^a,b^**	**Crude OR** **(CI 95%)**	**Adjusted OR** **(CI 95%) ^f^**
	**≤3 Episodes (*n* = 154)**	**>3 Episodes (*n* = 43)**			
***TNC* ^g^** **rs2104772 (T >A)**	***n* (%)**				
TT	38 (24.7)	8 (19.0)	0.32	1 ^c^	1 ^c^
TA	73 (47.4)	25 (59.5)		1.63 (0.67–3.95)	1.80 (0.71–5.60)
AA	43 (27.9)	9 (21.4)		0.99 (0.55–2.83)	1.05 (0.35–3.16)
TA + AA	116 (75.3)	34 (81.0)	0.36	1.39 (0.59–3.27)	1.52 (0.62–3.72)
***MMP3* ^h^** **rs591058 (C >T)**					
CC	54 (35.1)	5 (11.6)	**0.03**	1 ^c^	1 ^c^
CT	69 (44.8)	28 (65.1)		3.60 (1.28–10.09)	**4.14 (1.45–11.87)**
TT	31 (20.1)	10 (23.3)		2.79 (0.84–9.27)	**3.87 (1.15–13.02)**
CT + TT	100 (64.9)	31 (86.1)	**0.01**	3.35 (1.23–9.10)	**4.07 (1.46– 11.36)**

CI: confidence interval, ORs: odds ratios. ^a^ *p* ≤ 0.05 was obtained through the Chi-square test (Pearson’s *p*-value). ^b^ OR adjusted by age, sex, body mass index, nutritional guidance, post-training pain. ^c^ Reference group. ^d^ Genotyping successfully obtained from 396 athletes (tendinopathy = 196). ^e^ Genotyping successfully obtained from 385 athletes (control = 188). ^f^ OR adjusted by tendon pain and Training Exposure Index. ^g^ Genotyping successfully obtained from 196 athletes (>3 = 42). ^h^ Genotyping successfully obtained from 385 athletes (control = 188).

## Data Availability

The data presented in this study are available on request from the corresponding author.

## References

[1] Goes R.A., Lopes L.R., Cossich V.R.A., de Miranda V.A.R., Coelho O.N., Bastos R.D.C., Domenis L.A.M., Guimarães J.A.M., Grangeiro-Neto J.A., Perini J.A. (2020). Musculoskeletal injuries in athletes from five modalities: A cross-sectional study. BMC Musculoskelet Disord..

[2] Hopkins C., Fu S.C., Chua E., Hu X., Rolf C., Mattila V.M., Qin L., Yung P.S.-H., Chan K.-M. (2016). Critical review on the socio-economic impact of tendinopathy. Asia Pac. J. Sports Med. Arthrosc. Rehabil. Technol..

[3] Lopes L.R., Guimarães J.A.M., Amaral M.V.G., Pereira C.G., Wainchtock V.S., Goes R.A., Miranda V.A.R.D., Perini J.A. (2023). Genetic Polymorphisms in *COL1A2* gene and the Risk of Tendinopathy: Case-Control Study. Rev. Bras. Ortop..

[4] Lopes L.R., de Miranda V.A.R., Guimarães J.A.M., de Araujo Souza G.G., Wainchtock V.S., Grangeiro Neto J.A., de Araújo Goes R., Perini J.A. (2021). Association of *TNF-α-308G *>*A* polymorphism with susceptibility to tendinopathy in athletes: A case-control study. BMC Sports Sci. Med. Rehabil..

[5] Millar N.L., Silbernagel K.G., Thorborg K., Kirwan P.D., Galatz L.M., Abrams G.D., Murrell G.A., McInnes I.B., Rodeo S.A. (2021). Tendinopathy. Nat. Rev. Dis. Primers.

[6] Salles J.I., Lopes L.R., Duarte M.E.L., Morrissey D., Martins M.B., Machado D.E., Guimarães J.A.M., Perini J.A. (2018). Fc receptor-like 3 (-*169T*>*C*) polymorphism increases the risk of tendinopathy in volleyball athletes: A case control study. BMC Med. Genet..

[7] Salles J.I., Duarte M.E., Guimarães J.M., Lopes L.R., Vilarinho Cardoso J., Aguiar D.P., Machado Neto J.O., Machado D.E., Perini J.A. (2016). Vascular Endothelial Growth Factor Receptor-2 Polymorphisms Have Protective Effect against the Development of Tendinopathy in Volleyball Athletes. PLoS ONE.

[8] Parkinson J., Samiric T., Ilic M.Z., Cook J., Handley C.J. (2011). Involvement of proteoglycans in tendinopathy. J. Musculoskelet Neuronal Interact..

[9] Frantz C., Stewart K.M., Weaver V.M. (2010). The extracellular matrix at a glance. J. Cell Sci..

[10] Giantsis I.A., Diakakis N.E., Avdi M. (2020). High Frequencies of *TNC* and *COL5A1* Genotypes Associated With Low Risk for Superficial Digital Flexor Tendinopathy in Greek Indigenous Horse Breeds Compared With Warmblood Horses. J. Equine. Vet. Sci..

[11] Nie G., Wen X., Liang X., Zhao H., Li Y., Lu J. (2019). Additional evidence supports association of common genetic variants in *MMP3* and *TIMP2* with increased risk of chronic Achilles tendinopathy susceptibility. J. Sci. Med. Sport.

[12] Khoury L.E., Posthumus M., Collins M., van der Merwe W., Handley C., Cook J., Raleigh S.M. (2015). *ELN* and *FBN2* gene variants as risk factors for two sports-related musculoskeletal injuries. Int. J. Sports Med..

[13] Halper J., Kjaer M. (2014). Basic components of connective tissues and extracellular matrix: Elastin, fibrillin, fibulins, fibrinogen, fibronectin, laminin, tenascins and thrombospondins. Adv. Exp. Med. Biol..

[14] Nilsson-Helander K., Silbernagel K.G., Thomeé R., Faxen E., Olsson N., Eriksson B.I., Karlsson J. (2010). Acute achilles tendon rupture: A randomized, controlled study comparing surgical and nonsurgical treatments using validated outcome measures. Am. J. Sports Med..

[15] Zhang H., Hu W., Ramirez F. (1995). Developmental expression of fibrillin genes suggests heterogeneity of extracellular microfibrils. J. Cell Biol..

[16] Järvinen T.A., Józsa L., Kannus P., Järvinen T.L., Hurme T., Kvist M., Pelto-Huikko M., Kalimo H., Järvinen M. (2003). Mechanical loading regulates the expression of tenascin-C in the myotendinous junction and tendon but does not induce de novo synthesis in the skeletal muscle. J. Cell Sci..

[17] Sarasa-Renedo A., Chiquet M. (2005). Mechanical signals regulating extracellular matrix gene expression in fibroblasts. Scand. J. Med. Sci. Sports.

[18] Matsuda A., Hirota T., Akahoshi M., Shimizu M., Tamari M., Miyatake A., Takahashi A., Nakashima K., Takahashi N., Obara K. (2005). Coding SNP in tenascin-C Fn-III-D domain associates with adult asthma. Hum. Mol. Genet..

[19] Tashjian R.Z., Kim S.K., Roche M.D., Jones K.B., Teerlink C.C. (2021). Genetic variants associated with rotator cuff tearing utilizing multiple population-based genetic resources. J. Shoulder Elb. Surg..

[20] Kwan K.Y.C., Ng K.W.K., Rao Y., Zhu C., Qi S., Tuan R.S., Ker D.F.E., Wang D.M. (2023). Effect of Aging on Tendon Biology, Biomechanics and Implications for Treatment Approaches. Int. J. Mol. Sci..

[21] Munhoz F.B., Godoy-Santos A.L., Santos M.C. (2010). *MMP-3* polymorphism: Genetic marker in pathological processes (Review). Mol. Med. Rep..

[22] Raleigh S.M., van der Merwe L., Ribbans W.J., Smith R.K., Schwellnus M.P., Collins M. (2009). Variants within the *MMP3* gene are associated with Achilles tendinopathy: Possible interaction with the *COL5A1* gene. Br. J. Sports Med..

[23] Simunic-Briski N., Vrgoc G., Knjaz D., Jankovic S., Dembic Z., Lauc G. (2024). MMP3 single-nucleotide polymorphisms are associated with noncontact ACL injuries in competing high-level athletes. J. Orthop. Res..

[24] Lorenz D.S., Reiman M.P., Lehecka B.J., Naylor A. (2013). What performance characteristics determine elite versus nonelite athletes in the same sport?. Sports Health.

[25] Ainsworth B.E., Haskell W.L., Herrmann S.D., Meckes N., Bassett D.R., Tudor-Locke C., Greer J.L., Vezina J., Whitt-Glover M.C., Leon A.S. (2011). 2011 Compendium of Physical Activities: A second update of codes and MET values. Med. Sci. Sports Exerc..

[26] Larruskain J., Celorrio D., Barrio I., Odriozola A., Gil S.M., Fernandez-Lopez J.R., Nozal R., Ortuzar I., Lekue J.A., Aznar J.M. (2018). Genetic Variants and Hamstring Injury in Soccer: An Association and Validation Study. Med. Sci. Sports Exerc..

[27] Foster B.P., Morse C.I., Onambele G.L., Williams A.G. (2014). Variants within the *MMP3* gene and patellar tendon properties in vivo in an asymptomatic population. Eur. J. Appl. Physiol..

[28] Saunders C.J., van der Merwe L., Posthumus M., Cook J., Handley C.J., Collins M., September A.V. (2013). Investigation of variants within the *COL27A1* and *TNC* genes and Achilles tendinopathy in two populations. J. Orthop. Res..

[29] Kluger R., Burgstaller J., Vogl C., Brem G., Skultety M., Mueller S. (2017). Candidate gene approach identifies six SNPs in tenascin-C (*TNC*) associated with degenerative rotator cuff tears. J. Orthop. Res..

[30] Schlensog M., Ruehlmann A.C., Haeberle L., Opitz F., Becher A.K., Goering W., Buth J., Knoefel W.T., Ladage D., Meyer A. (2023). Tenascin-C affects invasiveness of EGFR-mutated lung adenocarcinoma through a putative paracrine loop. Biochim. Biophys. Acta Mol. Basis Dis..

[31] Dejnek M., Moreira H., Płaczkowska S., Barg E., Reichert P., Królikowska A. (2022). Effectiveness of Lateral Elbow Tendinopathy Treatment Depends on the Content of Biologically Active Compounds in Autologous Platelet-Rich Plasma. J. Clin. Med..

[32] Suhaimi S.A., Chan S.C., Rosli R. (2020). Matrix Metallopeptidase 3 Polymorphisms: Emerging genetic Markers in Human Breast Cancer Metastasis. J. Breast Cancer.

[33] Posthumus M., Collins M., van der Merwe L., O’cuinneagain D., Van Der Merwe W., Ribbans W.J., Schwellnus M.P., Raleigh S.M. (2012). Matrix metalloproteinase genes on chromosome 11q22 and the risk of anterior cruciate ligament (ACL) rupture. Scand. J. Med. Sci. Sports.

[34] Briški N., Vrgoč G., Knjaz D., Janković S., Ivković A., Pećina M., Lauc G. (2021). Association of the matrix metalloproteinase 3 (*MMP3*) single nucleotide polymorphisms with tendinopathies: Case-control study in high-level athletes. Int. Orthop..

[35] Kalembeyi I., Inada H., Nishiura R., Imanaka-Yoshida K., Sakakura T., Yoshida T. (2003). Tenascin-C upregulates matrix metalloproteinase-9 in breast cancer cells: Direct and synergistic effects with transforming growth factor beta1. Int. J. Cancer.

[36] Tremble P., Chiquet-Ehrismann R., Werb Z. (1994). The extracellular matrix ligands fibronectin and tenascin collaborate in regulating collagenase gene expression in fibroblasts. Mol. Biol. Cell.

[37] Valdivieso P., Toigo M., Hoppeler H., Flück M. (2017). T/T homozygosity of the tenascin-C gene polymorphism rs2104772 negatively influences exercise-induced angiogenesis. PLoS ONE.

[38] Siri A., Knäuper V., Veirana N., Caocci F., Murphy G., Zardi L. (1995). Different susceptibility of small and large human tenascin-C isoforms to degradation by matrix metalloproteinases. J. Biol. Chem..

